# Chancres of the Fingers

**Published:** 1891-02

**Authors:** 


### EDITORIAL.

### Chancres of the Fingers.

In another column of the REGISTER we called attention to the care of the hands, urging the necessity of properly cleaning the hands and finger nails before all operations upon the teeth.

In the Jan. 17th number of the *Medical Record* appears a leading article which was read before the section on Genito-Urinary Surgery of the New York Academy of Medicine, by R. W. Taylor, M.D., bearing the title which appears at the head of this article. The whole article with its superb illustrations would be an interesting publication for any dental journal, but we must rest content to publish in part that which most concerns the dental profession. Dr. Taylor says:

"Chancres of the fingers contracted in operations, examinations, and manipulations are not uncommon among obstetricians, surgeons, and midwives, and they are occasionally seen upon the hands of some luckless laymen as the result of their handling or lascivious fondling the infected genitals of some female. *They are also observed in dentists, contracted in operations on the mouths of syphilitic patients.* (Italics are ours).

They may be seated upon the finger pulp, or around the nail. Syphilitic finger-infection has been seen in children and women as a result of picking or dressing the hard chancre of a second party. Finger-chancres as a result of a bite by a syphilitic person are also occasionally seen. Chancres of the knuckles and fingers have been known to follow a blow delivered upon the mouth of a person suffering from specific lesions. These chancres are prone to form at some part of the nail margin, also on the sides and pulp of the finger, and along its continuity. In the greater number of cases they are due to the infection of a small fissure, a chap, an excoriation, or a cut, or to the lodgement of the virus between the nail and its tegumentary fold. Any skin disease of an inflammatory, or hyperplastic nature situated upon the fingers, or hands, may afford a port of entry for syphilitic infection.

The following extract is significant:

"The fact of the not uncommon occurrence of chancres upon the fingers of surgeons, physicians, and midwives, suggests to us the danger of syphilitic infection to their patients."

This, kind reader, is of ultra importance to consider even as a class of dentists. Whilst we do not believe that chancres may be found on the fingers of many dentists, yet they do occur, and may innocently have been infected. Dr. Taylor relates that physicians afflicted with syphilitic finger-infection have attended to their administrations to patients while ignorant of their own disease. Neisser says that he knows of four (4) infected assistants who keep on with their professional work notwithstanding their finger-chancres. It is strange that more infection does not take place.

Dr. Taylor further says: "It is evident that surgeons afflicted with syphilis are much less liable to infect their patients than obstetricians, particularly in these days of thorough asepsis and antisepsis."

Mark the following language:

"With the care that surgeons give to the perfect cleansing of their hands before operations, and with the fear in their minds (which is quite widespread) of receiving syphilitic or other infection through any lesion of continuity in the event of their having a chafe, a fissure, or any suspicious sore, or hyperplastic papule, nodule, or mass on the fingers, will, I think, cause them either to give up the operation, or take measures for the thorough protection of their fingers.

Then, again, the profuseness with which antiseptics, both liquid and pulverulent are used in operations, will undoubtedly do much to prevent syphilitic infection." Continuing he says:

"The period of greatest danger from finger infection by medical men is in the early days of the chancre, when it is small (like a chafe, a fissure, a mildly excoriated papule, or seemingly like a simple but toublesome hangnail, or a mild form of panaritium), and when its nature is not suspected. In this period, as a rule, it is very little, if at all, painful; it has not an angry or an inflamed look, but on the contrary, presents a benign, even insignificant appearance. In this stage, luckily, its secretion is very scant, and to this fact, therefore, many instances of immunity are undoubtedly due. But even under these circumstances the tissue-elements may be deposited from the infected finger upon a wound in the patient, and syphilis may ensue. Then, again, these seemingly mild lesions may issue to blood, which, if deposited upon an excoriated, exulcerated, or cut surface may convey the syphilitic poison. Though syphilitic blood infection is less prompt, active, and constant than infection from primary and secondary secretions and lesions, its danger should always be remembered, feared, and provided against."

"Chancres of the fingers in professional men usually cease to be dangerons to their clients when they have reached their full development."

"I have often wondered why many physicians and surgeons who have consulted me have remained so long in ignorance of the syphilitic nature of a finger chancre, and why it has not been recognized by their fellow practitioners to whom they have applied for diagnosis and treatment. I think it will be generally found when a surgeon, physician, obstetrician, or *dentist* has a chronic, indolent, aphlegmasic ulcer or nodule on his finger, particularly when near its tip, the leasion is, at least in the majority of instances, the forerunner of syphilis, especially if it has a bluish fungoid appearance, or the look of a piece of raw beef stuck on the finger end. Yet many think that they are the victims of some obscure blood-poisoning, or tuberculous infection."

The foregoing facts indicate very forcibly the duty of the dentist to refrain from touching his patients, making examinations, performing operations without a proper cleansing of the hands and finger-nails even though not infected himself he is liable to carry it from one patient to another.

Impervious court-plaster should always be possessed and ready to cover a cut of the finger, and a solution of bichloride of mercury is now found to be a necessity in a dental office. Accidentally an excoriation of the skin may occur, and sometimes a finger-cot may be used when deemed necessary in order to keep moisture out of the bruise. These and other things and methods may be devised to meet the wants on occasions where syphilis may be suspected. It is unnecessary to say that all napkins and rubber-dams used in such cases should be destroyed. W.

